# Oxytocin Levels Increase and Anxiety Decreases in Mothers Who Sing and Talk to Their Premature Infants during a Painful Procedure

**DOI:** 10.3390/children10020334

**Published:** 2023-02-09

**Authors:** Manuela Filippa, Maria Grazia Monaci, Carmen Spagnuolo, Massimiliano Di Benedetto, Paolo Serravalle, Didier Grandjean

**Affiliations:** 1 Swiss Center of Affective Sciences, Faculty of Psychology and Educational Sciences, University of Geneva, 1205 Geneva, Switzerland; 2 Department of Social Sciences, University of Valle D’Aosta, 11100 Aosta, Italy; 3 Maternal and Child Department, Parini Hospital, 11100 Aosta, Italy; 4 Department of Clinical Pathology, Parini Hospital, 11100 Aosta, Italy

**Keywords:** maternal voice, infant-directed singing, infant-directed speech, preterm infants, painful procedures, oxytocin, anxiety

## Abstract

(1) Background: Preterm infants spend their first weeks of life in the hospital partially separated from their parents and subjected to frequent potentially painful clinical procedures. Previous research has found that early vocal contact reduces infant pain perception while simultaneously increasing oxytocin (OXT) levels. The current study aims to assess the effect of maternal singing and speaking on mothers. (2) Methods: During a painful procedure over two days, twenty preterm infants were randomly exposed to their mother’s live voice (speaking or singing). Maternal OXT levels were measured twice: before and after singing, as well as before and after speaking. The anxiety and resilience responses of mothers were studied before and after the two-day interventions, regardless of the speaking/singing condition. OXT levels in mothers increased in response to both singing and speech. Concurrently, anxiety levels decreased, but no significant effects on maternal resilience were found. (3) Conclusions: OXT could be identified as a key mechanism for anxiety regulation in parents, even in sensitive care situations, such as when their infant is in pain. Active involvement of parents in the care of their preterm infants can have a positive effect on their anxiety as well as potential benefits to their sensitivity and care abilities through OXT.

## 1. Introduction

The impact of preterm birth and its consequences on an infant’s development [1] and parental mental health is a key concern for health policies in both low-income and high-income nations [2]. Preterm birth and length of hospitalization in neonatal intensive care units (NICUs) can be traumatic and stressful for parents during the newborn’s first weeks and months of life [3,4].

Separation of parents and newborns in the NICU has negative impacts not only on infants but also on parents. It is widely established that this early separation is a significant source of stress for newborns [5], with lasting effects on the infant’s immunological, neuroendocrine, and autonomic systems [6]. When parents exhibit a lack of responsiveness to an infant’s demands and social behaviors during important bonding periods due to stress or depression, the attachment process may be affected [7].

### 1.1. The Key Role of Oxytocin

If long periods of separation have deleterious effects on preterm infants, higher levels of contact soon after infant’s birth prompt a greater maternal production of OXT in both animals and humans [8,9]. OXT is a neuropeptide hormone crucial for social affiliations in humans [10]. Its functions are to promote prosocial behaviors [11], with neuroprotective effects on the development of infants and children. Specifically, in the period following birth, OXT regulates maternal behaviors [12] and supports positive social interactions [13]. OXT external administration increases affiliative behaviors in both parents and pupils [14], and endogenous OXT production in mothers is raised by somatosensory stimulation such as breastfeeding or sucking [15], especially in the neonatal period. Moreover, OXT is also involved in physiological responses to stress and pain [16]. In animal models, OXT has an analgesic effect because of its direct action on cerebral pain matrix structures [17,18]. In humans, it has effects on brain regions involved in the processing of pain; however, the exact mechanisms of action are not fully understood.

Although evidence for the analgesic benefits of OXT in humans is not as consistent as in animal models, there is rising evidence that OXT could lessen the perception or expression of pain [19], for instance, by modifying the emotional and cognitive components of the experience.

### 1.2. Maternal Anxiety and Resilience in the NICU

In general, parental anxiety is a construct that can have a deleterious effect on a child’s development, contributing to increased anxiety and depression levels in children.

Early separation between parents and newborns can also have long-term effects on parental mental health [2,4], with consequences on parent–infant interactions and, subsequently, on infants’ development [20].

Mothers often describe the experience of preterm birth and the subsequent hospitalization of their infant in the NICU as a profound source of anxiety [21] and as traumatic and overwhelming, making them feel powerless and confused in their maternal role [22,23]. The traumatic stress characterizing parent experiences during and after the NICU hospitalization is relevant [24], and NICU women exhibit greater signs of anxiety, stress, and depression than mothers of full-term, healthy infants [25].

Moreover, the NICU sound environment, with its unpredictable noises and continual alarms, is well-known to have detrimental short- and long-term consequences on the behavior and development of such fragile preterm infants [26] as well as on parental anxiety, stress, and depression [27]. Parents experience the preterm birth and NICU stay as difficult events [28,29,30], and early individualized interventions have been developed to reduce or eliminate anxiety in parents of infants admitted to the NICU [31].

However, it is difficult to determine why one individual is badly affected by a stressful and traumatic event, such as premature birth, while another is able to react and provide the necessary support for the growth of the newborn. Some scholars have proposed the concept of resilience, focusing on defining its essential characteristics and attempting to quantify this capacity.

Recently, the concept of maternal resilience has been defined as the capacity to adapt and/or adjust to the impacts of traumatic events in order to live a complete and meaningful life [32]. Parents, during hospitalization, tend to develop resilience and strength in order to become advocates for their infants and to promote the infants’ health, and one of the successful programs for supporting them in this stressful and traumatic experience, adopted in an increasing number of NICUs, is the parent’s active involvement in a family-centered care approach [33,34].

### 1.3. Early Protective Actions Involving Parents in the NICU: Early Vocal Contact

Everyday hospital living exposes preterm infants to painful experiences [35,36]. Despite the efforts of medical and nursing staff in developing and implementing family-based care, very often, parents are not present to support newborns during painful experiences.

Parental presence is a non-pharmacological protective condition as it is associated with a reduction in pre-procedural pain in preterm infants [37]. The effectiveness of breastfeeding and skin-to-skin contact in decreasing the pain response in preterm infants is well tested [38,39], including at the cortical level [40].

An active and participative presence of parents in the NICU is nowadays considered crucial for the physical and psychological well-being of infants and parents [41,42]. When parents engage with their preterm newborn, they can regulate the infant’s state and behavior via skin-to-skin contact and vocalizations. Early vocal contact is an early intervention that engages parents in vocal, emotional, and meaningful interactions with their preterm infants during NICU admission [43].

In particular, the maternal voice promotes the physiological stability of the preterm infant, with a considerable reduction in critical episodes such as bradycardia, apnea, and hypoxia and a smoother transition to quiet waking states [44]. Early exposure to adult voices, not just parental voices, is also significantly correlated with better cognitive and language development at 7 and 18 months [45].

On the contrary, if a newborn baby is left alone in its room for a long time, without the possibility of hearing the voice of its parents or other adults, it runs the risk of being deprived of the linguistic stimulus that is so important for its cerebral development, with long-term negative effects on its neurodevelopment [46].

Filippa and colleagues used microanalytic analysis techniques to assess and describe the regulatory effect of maternal and paternal vocalizations on the behavioral organization of preterm infants [47,48]. During early vocal interaction with their mother, infants increase two specific behaviors, self-touch gestures and eye-opening [47]. Eye-opening and smiling appear to be of particular importance to parents, who adapt their voice to the infant’s behavior by increasing the pitch and intensity variability of infant-directed speech and songs [49]. In addition, when the infant opens his eyes and smiles, the mother responds immediately by modulating her voice, which is perceived by other adults as richer in emotion during the interaction [50,51].

The present data are part of previous research in which Filippa et al. [19] demonstrate that the mother’s speech has a positive effect on the pain expression of preterm infants. This is evidenced by a significant drop in the Preterm Infant Pain Profile [52] score, which is a cluster of physiological and behavioral measures commonly used in clinical practice and research to assess pain in preterm newborns. Concomitantly, OXT levels significantly increase when the mother is talking, but only marginally for the singing condition.

In the last decades, it has become increasingly important to validate early interventions that actively involve the participation of parents alongside their infants in the NICU, even during the overmentioned potentially stressful situations, to evidence the potential mechanisms underpinning the protective actions of parents. What is less known, in fact, is the effect of parental interventions during painful procedures on parental mental health. The overall aim of the present work is to investigate how infant-directed singing and speaking modulate OXT levels and the anxiety response in mothers during their infant’s painful procedure. The aim is achieved by comparing OXT and maternal anxiety and resilience levels in the singing and speaking conditions, before and after the interventions.

The present results may shed new light on the effects of human proximity as a protective experience against pain and anxiety in at-risk conditions.

## 2. Materials and Methods

Study design and participants. The trial was carried out at a hospital in Aosta in a level II NICU (Italy), where newborns are admitted if they are more than 29 weeks in gestational age and do not have any critical pathologies or syndromes. A senior clinician evaluated newborns for eligibility; 21 of the 68 preterm babies delivered in the NICU hospital between January 2018 and April 2019 met the inclusion criteria, and they were invited to enroll. Infants were included if they were more than 29 weeks in gestational age (GA), weighed more than 1000 g, and had a stable health condition. Their mean GA at birth was 32.7 weeks GA (SD = 9.6 days); their mean birthweight was 2042 g (SD = 392.5 g). Brain ultrasound examinations are not routinely performed in this NICU, and none of the newborns included in this study underwent neuroimaging exams.

Mothers having a history of drug or alcohol abuse or mental illness were not included.

Twenty mothers volunteered to participate; 95 percent of the sample was Italian, and 41.7% held advanced educational degrees (that is, they at least graduated). Their ages ranged from 23 to 34 years, with a mean of 29.2 years.

In the sample, 90% of the mothers were first-time mothers. Primiparous mothers have been shown to have higher levels of anxiety than non-primiparous mothers, as well as greater physiological reactivity to their babies [53]. The details of the maternal deliveries have also been reported as a descriptor of the population characteristics for the impact that a cesarean delivery can have on maternal anxiety [54]. The mothers’ characteristics are summarized in Table 1.

The official Hospital Ethical Committee of Aosta reviewed and approved the study (I.C. n. 90.513; date of approval: 20 October 2017).

Procedure. The procedure was also reported in a previous article [19]. Each newborn was tested on two occasions. During the intervention, mothers were asked to speak (day 1) or sing (day 2) to their preterm infants in the incubators for 5 min before and after the heel-prick procedure. The singing and speech were performed in random order. All of the mothers stopped singing and speaking 5 min after the painful procedure, which lasted no longer than one minute. The speaking and singing interventions were carried out over two days in the first days of extra-uterine life when preterms were in their first days of post-natal age (mean = 3 days of post-natal age, range 1–8 days). Newborns’ mean GA at the first intervention day was 34.8 weeks of GA, and they weighed 2264 g (mean value). The study investigator registered new patients and obtained treatment arm assignments using a secure web-based randomization system. The order of the singing and speaking [19] was randomly selected and assigned to two different days (i.e., on day one, the mothers sang, and on day two, they intervened with the speech, or vice versa).

During the interventions, moms were instructed not to touch the newborn but to pay close attention to his or her responses and adjust their voices accordingly. An attending nurse was present for all procedures. We instructed mothers not to exceed the recommended sound pressure levels [55] to avoid overstimulation. In order to ensure that the mother’s voice was heard by the newborn (i.e., that it was 10 dBA louder than the background noise) [56], background noise levels were measured with a calibrated sound level meter (Voltcraft Phonometer SL-10; Conrad Electronic, Hirschau, Germany) in the room where the procedure was taking place, inside the incubator, and 20 cm from the infant’s head. This measurement was assessed before each intervention.

Lastly, the mothers were asked to open the incubator’s window, talk or sing through it, and keep their heads about 20 cm away from the baby’s head. The researcher always made sure that the right position was in place.

There were no other specific instructions given to the mothers, who were free to use their intuitive parenting behaviors in close proximity to the babies and choose the contents of their speech and singing. These values were not subjected to any further analysis.

### 2.1. Outcome Measures

OXT measure. The levels of maternal OXT were measured twice: once before and once after the singing, as well as before and after speaking. Using an absorbent device [57], mothers’ saliva samples were collected, centrifuged, and kept until analysis. For each of the two interventions, singing and speaking, saliva samples were obtained twice, 5 min before and after the heel-prick procedure, for a total of four saliva samples. Salivary OXT concentrations [58] were measured by radioimmunoassay (RIAgnosis, Munich, Germany).

For each sample, 300 μL of saliva was evaporated (SpeedVac, Thermoscientific Inc., Waltham, MA, USA), followed by the addition of 50 μL of assay buffer and 50 μL of antibodies. After a 60 min preincubation period, an additional 10 μL of a 125 I-labelled tracer (PerkinElmer, Waltham, MA, USA) was added to the samples, which were then incubated at 4 °C for three days. The detection limit was fixed at the 0.5 pg/sample range, with typical displacements of 20–25% at 2 pg, 60–70% at 8 pg, and 90% at 32 pg of the standard neuropeptide. Both the intra- and inter-assay variances were less than 10%.

Anxiety measure. The anxiety responses of mothers were examined solely before and following the two-day interventions, independent of the speaking/singing condition. Anxiety levels were measured with the Italian version of the State-Trait Anxiety Inventory [59] (Italian version, [60]). STAI questionnaires were collected two times, before and after the two days of intervention (singing and speaking). The STAI is a widely used self-report instrument distinguishing between dispositional (trait) and transitory (state) types of anxiety. State anxiety can be described as an unpleasant emotional state or condition incorporating feelings of tension, nervousness, and worry, and trait anxiety can be described as a relatively stable personality trait. Each subscale consists of 20 items that are rated on a 4-point Likert scale. Responses were summed up, and higher scores reflect more state anxiety (range 20–60). The Cronbach’s alphas for the state subscale, notwithstanding the small size of the present sample, were very good (0.93 at pre and 0.94 at post).

Resilience measure. Mothers’ resilience responses were examined only before and after the two-day interventions, regardless of the speaking/singing condition. Wagnild and Collins [61] developed a 25-item self-report scale to assess resilience. As an extension of the original 25 questions, they created a 14-item scale. On a 7-point Likert scale ranging from 1 (strongly disagree) to 7 (strongly agree), participants were asked to rate their agreement or disagreement with each statement (strongly agree). The lowest possible score on the 14-item scale is 14, and the highest possible score is 98. According to Wagnild et al., a score of 91 indicates a high level of resilience; our scores ranged from 64 to 92, with the majority of the sample scoring higher than 83. Cronbach’s alpha for the subscale was acceptable (0.81 at pre and 0.86 at post).

### 2.2. Data Analysis

To determine variations in the mother’s OXT levels before and after the interventions, a 2 (Condition: Speaking and Singing) × 2 (Time: Pre and Post) repeated-measures analysis of variance (ANOVA) with two within-subject factors was performed, followed by a post hoc *t*-test.

Second, regardless of the speaking/singing condition, a paired *t*-test was performed on maternal anxiety levels and resilience before and after the two-day interventions (see limitation paragraph in the Discussion section).

## 3. Results

**Figure 1:**
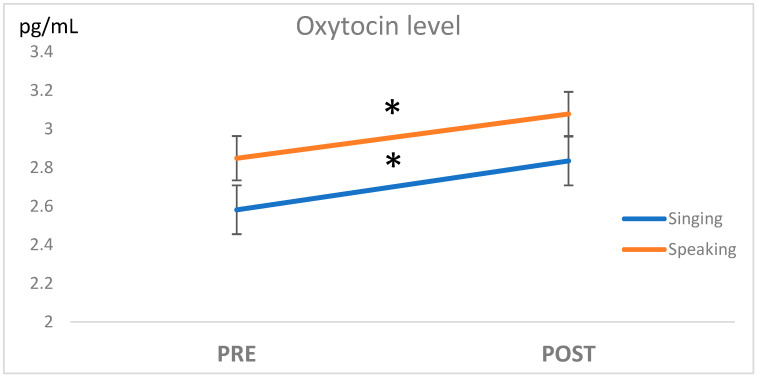
A significant increase was observed in maternal OXT mean levels (pg/ μL) after (Post) the intervention in both Singing and Speaking conditions. * *p* < 0.05.

Before the maternal interventions, the maternal OXT levels in the Singing and Speaking conditions were not significantly different (*p* = 0.43, Figure 1).

A significant effect of the intervention was observed (F_(18,1)_ = 8.7, *p* = 0.008, η^2^= 0.327; a participant who had one missing value on the OXT level was removed from the ANOVA).

A post hoc test with a paired *t*-test confirmed that the level of OXT was significantly different between the Pre and Post measures, both for the Singing condition (*t*-test (18) = 2.82, *p* = 0.011) and for the Speaking condition (*t*-test(19) = 2.43, *p* = 0.025), while there were no significant differences between Speaking and Singing conditions after the interventions (*t*-test (19) = 0.79, *p* = 0.44).

**Figure 2:**
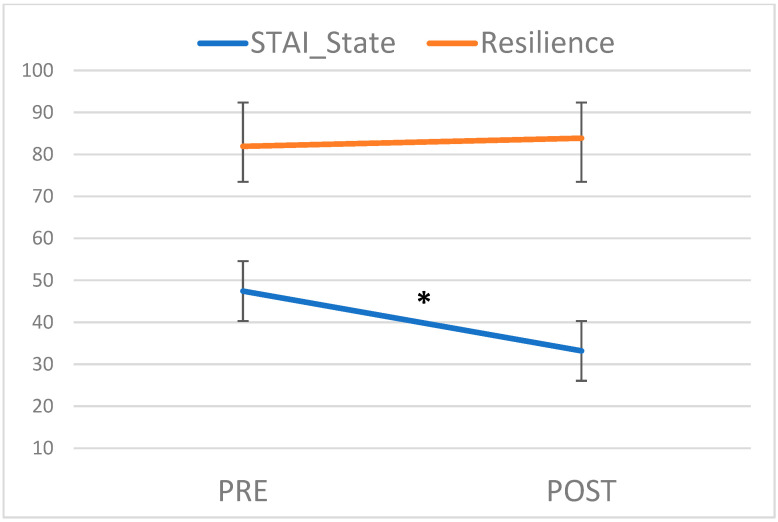
A significant decrease was observed in the STAI state after the mothers’ singing and speaking interventions. No significant changes were evidenced for the resilience levels. * *p* < 0.05.

A significant effect of the intervention was observed (*t*-test (19) = 4.56, *p* < 0.001) on anxiety levels, which were significantly lower after the Singing and Speaking interventions (Post) when compared to the Pre measures (see Figure 2; M = 47.45, SD = 8.27 vs. M = 33.20, SD = 10.22). The STAI scores anxiety levels ranged from 20 to 60, with 20 being the lowest and 60 being the highest. A score of 41 is thought to be the cut-off for determining an optimal level of anxiety [62]. Mothers’ anxiety mean scores were above the cut-off level before the two-day interventions and below it after the two-day interventions.

In a *t*-test comparison, no significant differences in any of the previously reported study variables emerged between women who gave birth by spontaneous vaginal delivery or by cesarean section.

No significant effect of the intervention was observed on the resilience score (*t*-test (18) = 1.44, *p* = 0.17; M_PRE_ = 82.0, SD_PRE_ = 6.9; M_POST_ = 83.9, SD_POST_ = 6.5).

The means and standard deviations of the study variables, OXL levels, and anxiety and resilience measures are presented in Table 2.

Significant correlations (*p* < 0.05) were found between all the OXT values before and after both the Singing and Speaking conditions. Anxiety levels, measured with the STAI before and after the two-day interventions, were positively correlated; STAI pre and post were positively correlated with resilience, but only in the values collected before the two-day interventions (Resilience_pre, see Table 3).

There were significant positive correlations (*p* < 0.05) between all of the OXT values before and after both the Singing and Speaking conditions. Anxiety levels were correlated with the STAI before and after the two-day interventions; STAI pre and post were correlated with resilience, but only in the values collected before the two-day interventions.

The correlations for all the study variables are presented in Table 3.

## 4. Discussion

Maternal levels of OXT were measured in mothers before and after singing and speaking during their preterm infant’s painful procedures. Maternal anxiety and resilience were measured only before and after the two-day interventions, regardless of the speaking/singing condition.

Preterm infants are routinely exposed to heel-prick painful procedures during hospitalization, often in the absence of a parent. In the present study, we demonstrate not only that maternal singing and speaking during an infant’s painful procedure is feasible but that it is also beneficial for mothers, who increase their OXT levels and, concomitantly, decrease their anxiety levels.

OXT is a neuropeptide playing a crucial role in human and animal affiliations [63], and it modulates sensory processing and pain perception, acting as a potent analgesic [17]. Its endogenous increase, as demonstrated in the present study, can induce an effect of anxiety protection similar to the one caused by OXT injections [64]. Concomitantly, a reduction in maternal anxiety in the neonatal period is of paramount importance for preventing later problems of attachment and bonding between parents and infants [65].

The present results confirmed previous studies reporting that maternal live singing and speaking to preterm babies have positive effects not only on infants but also on parents [47,48]. Infant-directed singing has a beneficial effect on parents [66], and it supports interindividual synchronization, affecting heart rate and breathing [67]. Infant-directed singing and speech interaction could then have a direct effect on maternal anxiety, modulated by OXT release.

We expected that maternal singing and speaking could sustain resilience in parents who could sustain their infants in difficult, painful conditions, but no significant results supported our hypothesis. This outcome may be attributable to the specificity of the resilience measurement scale. In actuality, the response scores are in the highest percentiles, with the majority of the sample scoring over 83, leaving little opportunity for improvement following the intervention.

As expected, the correlation analyses between the study variables reveal a pattern of significant and moderate/high connection between the various oxytocin measures, particularly in the pre–post measurements. Similarly, and predictably, the pre- and post-resilience measures have a significant and high correlation.

Surprisingly, in addition to the significant and moderate correlations between the pre- and post-STAI states, only the post-STAI state has significant, albeit modest, negative correlations with both pre- and post-resilience.

We expected resilience in parents of preterm infants to be negatively correlated with anxiety even before the intervention based on previous research in a similar population [68]. On the contrary, this negative correlation only becomes significant after a two-day intervention. This finding supports the hypothesis that future research should investigate the concept of resilience but with more sensitive instruments tailored to the unique circumstances of parents and the trauma of preterm birth. Furthermore, in order to determine whether or not patients are at risk for low resilience, researchers should consider a broader definition of resilience that includes not only self-reported questionnaires but also a more complex investigation of multiple social indicators, such as lack of social contacts, poor family functioning, and low cohesion among family members.

A significant limitation of the study is that the anxiety and resilience questionnaires were only administered once after each session, thus making it impossible to compare anxiety levels following the speech or song intervention. We reasoned that the psychological constructs of resilience and anxiety better demonstrate the impact of early intervention after the entire intervention cycle, so we aimed to evaluate the overall impact of the two-day intervention without making a distinction between singing and speaking. However, we could also hypothesize that the two styles of voice engagement also have different consequences on the parents; it is anticipated that singing regulates the infant’s parasympathetic activity, as indicated by heart rate variability characteristics [69], and speaking is expected to increase active states in preterms [44] and to effectively reduce pain [19] during routine heel-prick procedures. To disentangle the distinct effects of the two contact modalities, future research should measure anxiety and resilience after each singing and speaking session.

## 5. Conclusions

Maternal singing and speech increase OXT levels and decrease anxiety in mothers during their preterm infant’s painful procedures. Similarly, in a previous study, we demonstrated that OXT endogenous production increases in infants as well [19]. OXT seems to be, thus, a reliable mechanism both for pain and anxiety protection, respectively, in infants and in parents.

The present results confirm that the release of OXT can be conditioned to emotional states, in our case, to anxiety, and the actions of this peptide may provide an additional explanation for the short- and long-term benefits of positive contact and vocal experiences in the NICU.

## Figures and Tables

**Table 1:** Mothers’ demographics.

Mode of delivery	Spontaneous vaginal delivery (40%) Cesarean section (60%)
Citizenship	Italian (95%)
Primiparous	Yes (90%)
Age	M = 29.2 (SD = 4.62)

**Table 2:** The study variables’ means and standard deviations.

	OXT_Singing (pg/μL)		OXT_Speaking (pg/μL)		STAI_State Range 20–60		Resilience Range 14–98	
	Pre	Post	Pre	Post	Pre	Post	Pre	Post
M	2.6	2.8	2.8	3.0	47.5	33.,2	82.0	83.9
SD	1.3	1.4	1.7	2.0	8.3	10.2	6.9	6.5

**Table 3:** Pearson’s correlation coefficients for the variables under consideration. * *p* < 0.05.

	1	2	3	4	5	6	7	8
1. OXT_Singing_Pre	─	0.97 *	0.58 *	0.56 *	−0.03	0.11	−0.15	−0.04
2. OXT_Singing_Post		─	0.67 *	0.67 *	−0.01	0.11	−0.17	0.01
3. OXT_Speaking_Pre			─	0.99 *	−0.01	−0.04	−0.31	−0.22
4. OXT_Speaking_Post				─	−0.04	−0.06	−0.27	−0.19
5. STAI_State_Pre					─	0.40 *	0.08	−0.49 *
6. STAI_State_Post						─	0.36	−0.49 *
7. Resilience_Pre							─	0.88 *
8. Resilience_Post								─

## Data Availability

The data presented in this study are available on request from the corresponding author.
